# Enhanced Oxide Ion Conductivity by Ta Doping of Ba_3_Nb_1*–x*_Ta_*x*_MoO_8.5_

**DOI:** 10.1021/acs.inorgchem.2c03943

**Published:** 2023-01-17

**Authors:** Brent Sherwood, Eve J. Wildman, Ronald I. Smith, Abbie C. Mclaughlin

**Affiliations:** † Department of Chemistry, University of Aberdeen, Meston Walk, AberdeenAB24 3UE, U.K.; ‡ ISIS Facility, STFC Rutherford Appleton Laboratory, Harwell Campus, DidcotOX11 0QX, U.K.

## Abstract

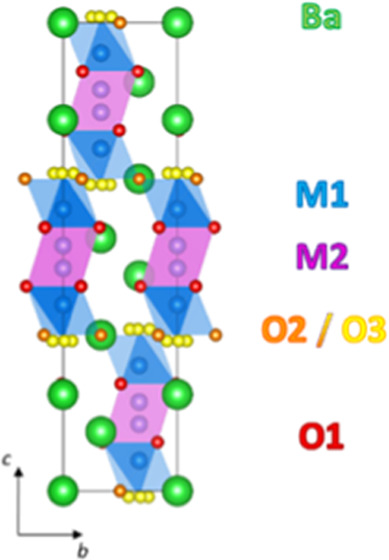

Significant oxide ion conductivity has previously been reported for the Ba_3_M′M″O_8.5_ family (M′ = Nb^5+^, V^5+^; M″ = Mo^6+^, W^6+^) of cation-deficient hexagonal perovskite derivatives. These systems exhibit considerable structural disorder and competitive occupation of two distinct oxygen positions (O3 site and O2 site), enabling two-dimensional (2D) ionic conductivity within the *ab* plane of the structure; higher occupation of the tetrahedral O3 site vs the octahedral O2 site is known to be a major factor that promotes oxide ion conductivity. Previous chemical doping studies have shown that substitution of small amounts of the M′ or M″ ions can result in significant changes to both the structure and ionic conductivity. Here, we report on the electrical and structural properties of the Ba_3_Nb_1–*x*_Ta_*x*_MoO_8.5_ series (*x* = 0.00, 0.025, 0.050, 0.100). AC impedance measurements show that substitution of Nb^5+^ with Ta^5+^ leads to a significant increase in low-temperature (<500 °C) conductivity for *x* = 0.1. Analysis of neutron and X-ray diffraction (XRD) data confirms that there is a decrease in the M1O_4_/M1O_6_ ratio upon increasing *x* from 0 to 0.1 in Ba_3_Nb_1–*x*_Ta_*x*_MoO_8.5_, which would usually coincide with a lowering in the conductivity. However, neutron diffraction results show that Ta doping causes an increase in the oxide ion conductivity as a result of longer M1–O3 bonds and increased polyhedral distortion.

## Abstract 2

The solid solution Ba_3_Nb_1−*x*_Ta_*x*_MoO_8.5_ has been synthesized with *x* up to 0.1. Results show that when *x* = 0.1, there is an expansion in the M1−O3 bond length, which leads to enhanced oxide ion conductivity.

## Introduction

Solid-state oxide ion conducting materials play an important role as electrolytes in electrochemical conversion devices such as solid oxide electrolyzer cells (SOECs)^1^ and solid oxide fuel cells (SOFCs).^2^ One of the main limiting factors of these devices is the low ionic conductivity of the electrolyte at room temperature. To achieve the sufficiently high oxide ion conductivities required for real-world operation (>10^–2^ S cm^–1^), temperatures of between 500 and 1000 °C are currently necessary. However, such high operating temperatures result in long start-up times, reduced efficiency, and lower operational lifetimes. Therefore, research into intermediate temperature (300–600 °C) solid electrolytes has become a prominent field, leading to the development of highly conductive material families such as LAMOX (La_2_Mo_2_O_9_) and BIMEVOX (Bi_4_V_2_O_11_), as well as doped lanthanum gallates (LSGM) and sodium bismuth titanates (NBT).^3−6^ A structural motif common to these ionic conductors is a flexible lattice, in which the cations are distorted from their ideal positions and oxygen defects (vacancies and interstitials) are present. Although these materials possess substantially higher ionic conductivities, the presence of easily reduced species such as Mo^6+^ and Bi^3+^ precludes their use in fuel cells due to the required operating conditions (low oxygen partial pressures) at the fuel electrode.^7,8^ In addition, large-scale production of LSGM and NBT is hindered by the formation of poorly conducting secondary phases and precise stoichiometric requirements, respectively.^9,10^ Furthermore, the increasing demand for low-carbon energy generation solutions may pose future supply risks for elements commonly used in solid electrolyte production; much like the proliferation of electric vehicles is massively increasing the demand for lithium, so too could widespread fuel cell adoption cause many of the required rare-earth elements see demand rise beyond supply.^11,12^ This necessitates the diversification of energy production and storage technologies, ideally using materials composed of earth-abundant elements.

Hexagonal perovskite derivatives have recently been shown to exhibit significant oxide ion and/or proton conductivity.^13−16^ Ba_3_NbMoO_8.5_ exhibits a bulk conductivity of 2.2 × 10^–3^ S cm^–1^ at 600 °C and good stability over a wide range of oxygen partial pressures,^17^ similar to commercial solid-state oxide ion conductors such as YSZ.^18^ Ba_3_NbMoO_8.5_ crystallizes in a hybrid of the 9R perovskite (A_3_B_3_O_9_) and palmierite (A_3_B_2_O_8_) structures. The 9R perovskite consists of nine AO_3_ layers stacked along the *c*-axis in the order (*hhc*)_3_, with trimers of face-sharing BO_6_ octahedra connected via corner-sharing. The palmierite structure is a cation-deficient derivative of the 9R perovskite, arising from the replacement of the cubic AO_3_ layers with oxygen-deficient AO_2_ layers, thereby forming isolated tetrahedral units separated by empty octahedral sites.

**Figure 1:**
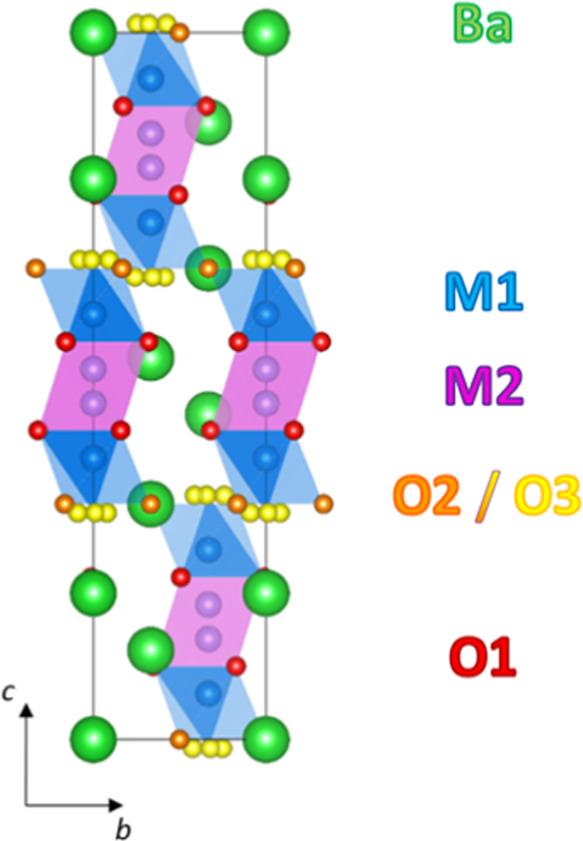
Average hybrid crystal structure of Ba_3_NbMoO_8.5_ viewed along the *a*-axis. Blue and light blue polyhedra represent the average M1O*_x_* units created by partial occupation of the O3 and O2 sites, respectively. To account for disorder, the M2 and O3 positions are split from their ideal sites.

The average hybrid model of Ba_3_NbMoO_8.5_ is shown in Figure 1. Nb^5+^ and Mo^6+^ are distributed across two crystallographic sites, M1 and M2. A single-crystal X-ray diffraction (XRD) study highlighted that the M1 and M2 positions are partially occupied due to their close proximity to one another (∼1.6 Å), thus making simultaneous occupation unfavorable.^19^ Hence, only two cation sites per hybrid stack may be occupied at once. The 1:1 ratio of Nb^5+^ to Mo^6+^ leads to a nonstoichiometric oxygen content, where partial occupation of the O2 and O3 sites within the palmierite-like layers (of average composition [BaO_2.5_]) creates a variable coordination environment surrounding the M1 site (M1O_*x*_); a recent pair distribution function (PDF) study has confirmed the existence of local 4-, 5-, and 6-fold M1 geometries.^20^ The resulting disordered distribution of intrinsic oxygen vacancies and available oxygen sites creates an uninterrupted two-dimensional pathway for the migration of oxide ions along the palmierite-like layers.^21^

The flexibility of the M1 site for multiple coordination environments is a key structural motif; the highest ionic conductivity is observed where tetrahedral M1O_4_ geometry is prevalent in the palmierite-like layer. A variable-temperature neutron diffraction study of Ba_3_MoNbO_8.5_ highlighted a distinct structural evolution between 25 and 600 °C.^22^ As the temperature increases, the relative average occupancy of the O2/O3 sites shifts in favor of the average O3 site above 300 °C; hence, the ratio of local M1O_4_ tetrahedra to M1O_6_ octahedra increases. This is accompanied by a simultaneous increase in oxide ion conductivity due to the proliferation of lower-energy migration pathways. This is supported by PDF analysis, which also shows an increase in the percentage of local tetrahedral M1O_4_ units at high temperatures, with a corresponding decrease in the percentage of M1O_6_ octahedra.^20^

A recent study attributes this structural rearrangement to the loss of absorbed water from the palmierite-like layers.^23^ The presence of absorbed water has been well documented for the hexagonal perovskite derivatives Ba_3_NbMoO_8.5_ and Ba_7_Nb_4_MoO_20_.^13,24^ Density functional theory (DFT) calculations have shown that if a proton points directly at the M1 site, the repulsion forces displacement of the metal ion onto the empty M2 site, creating the cationic disorder.^24^ Upon heating, these protons are removed from the structure (as water), allowing displaced M2 cations to return to the M1 sites, resulting in the observed redistribution of the oxygen population and an increase of the highly mobile O3 sites.

Doping studies have shown that the identity and oxidation state of the metal cations influence the occupancy of the O2/O3 sites. Partial substitution of Nb^5+^ for V^5+^ increases the average number of lower coordination M1O_*x*_ units, thereby improving the conductivity.^16^ Conversely, substitution of Mo^6+^ for either Nb^5+^ or W^6+^ causes a decrease in the average number of lower coordination M1O_*x*_ units and consequently lowers the conductivity.^25−27^ These results align with the preference of V^5+^ and Mo^6+^ for lower coordination environments.^28^ Ge^4+^ possesses a similar affinity for tetrahedral geometry. A recent doping study found that Ba_3_Mo_1+*x*_Nb_1–2*x*_Ge_*x*_O_8.5_ (*x* = 0.2) exhibits a bulk conductivity twice that of the parent compound.^29^ The conductivity of such materials may be further enhanced by displacement of the M1 site away from the mobile O2/O3 oxygens and distortion of the polyhedra by the second-order Jahn–Teller effect; both structural and electronic effects, respectively, are mutually supportive.^22,30^

Ta doping has previously been shown to enhance the ionic conductivity and/or stability of several crystal systems and significantly increase their resilience to reducing atmospheres.^31−33^ Here, we report the electrical and structural properties of the Ba_3_Nb_1–*x*_Ta_*x*_MoO_8.5_ series (*x* = 0.00, 0.025, 0.050, 0.100).

## Experimental Section

Ba_3_Nb_1_–*x*Ta_*x*_MoO_8.5_ (*x* = 0.00, 0.025, 0.050, 0.100) compounds were prepared by solid-state reaction of stoichiometric amounts of BaCO_3_ (99.999%, Sigma-Aldrich), Nb_2_O_5_ (99.98%, Sigma-Aldrich), Ta_2_O_5_ (99.98%, Sigma-Aldrich), and MoO_3_ (99.98%, Sigma-Aldrich). These starting materials were ground using a mortar and pestle until homogeneous. The resulting powders were pressed into a pellet and calcined in an alumina crucible at 900 °C for 12 h to decarbonate. The pellets were then re-ground, re-pelleted, and heated at 1100 °C for 48 h before being cooled to room temperature at a rate of 5 °C min^–1^. The latter grinding and heating step was repeated until phase-pure products were obtained.

Room-temperature X-ray powder diffraction (XRD) patterns were collected on a PANalytical Empyrean powder diffractometer equipped with a Cu Kα tube. Data were recorded in the range 10° < 2θ < 100°, with a step size of 0.013°.

Time-of-flight (TOF) neutron powder diffraction data were collected at 20 °C on the Polaris diffractometer^34^ at the ISIS Neutron and Muon Source, U.K. A 5 g sample of Ba_3_Nb_0.9_Ta_0.1_MoO_8.5_ was loaded into a cylindrical vanadium can of 8 mm diameter, and data were collected for a total of 2 h. Rietveld refinement was performed using the GSAS/EXPGUI package.^35,36^

Scanning electron microscopy (SEM) images were collected using a Carl Zeiss GeminiSEM 300 with an XMax 80 detector and an AZtecHK EBSD analysis system with a Nordlys Nano EBSD camera (Oxford Instruments Ltd.). Energy-dispersive X-ray (EDX) spectroscopy data were mapped using the Oxford INCA X-ray microanalysis software to identify the elemental composition of the sample. Samples were mounted on a stub and coated with a thin layer of carbon.

The PIEFACE software package was used to fit the distorted coordination polyhedra using the minimum bounding ellipsoid method.^37^ The standard deviation, σ(*R*), of the three principal ellipsoid radii (*R*_1_ ≥ *R*_2_ ≥ *R*_3_) was used to quantify the polyhedral distortion of the system. The value *S* quantifies the degree to which the ellipsoid is prolate (1 ≥ *S* > 0), spherical (*S* = 0), or oblate (0 > *S* ≥ −1).

The electrical properties of the Ba_3_Nb_1–*x*_Ta_*x*_MoO_8.5_ (*x* = 0.00, 0.025, 0.050, 0.100) series were measured by AC impedance spectroscopy using a Solartron 1260 impedance analyzer in the frequency range of 0.1 Hz to 1 MHz with an applied alternating voltage of 0.1 V. Measurements were performed on platinum-coated pellets of approximately 1 mm thickness and 10 mm diameter possessing a density 95% of the theoretical density. Measurements were taken every 15 °C (allowing 2 h of equilibration at each temperature step) upon cooling from 600 °C in a sealed tube furnace. Measurements were taken in a dry gaseous atmosphere (*p*H_2_O < 10^–4^ atm), obtained by flowing compressed air through a column of a commercial desiccant (Drierite).

## Results and Discussion

### Characterization of Ba_3_Nb_1–*x*_Ta_*x*_MoO_8.5_ (*x* = 0.00, 0.025, 0.050, 0.100)

Laboratory powder X-ray diffraction (XRD) confirmed all of the as-prepared Ba_3_Nb_1–*x*_Ta_*x*_MoO_8.5_ (*x* = 0.00, 0.025, 0.050, 0.100) samples to be phase-pure. All phases could be indexed to the unit cell with space group *R*3̅*mH*, in agreement with previous reports.^16,17,38^ The average stoichiometry of each sample was confirmed by EDX measurements (Table S1). SEM micrographs showed irregular shaped grains with sizes ranging from ∼5 to 10 μm (Figure S1) with a similar microstructure to the Ba_3_M′M″O_8.5_ phases reported previously. Synthesis of the composition *x* = 0.125 and higher *x* was attempted but could not be made phase-pure (the impurity was identified as Ba_6_Nb_3_O_13.5_), suggesting this is the limit of the solid solution.

Using high-resolution XRD data, Rietveld refinement of the *R*3̅*m H* model for the Ba_3_Nb_1–*x*_Ta_*x*_MoO_8.5_ series was performed to investigate the structural changes effected by Ta substitution. The model of the parent compound Ba_3_NbMoO_8.5_^16^ refined from neutron diffraction data was used as the starting point for each composition of the Ba_3_Nb_1–*x*_Ta_*x*_MoO_8.5_ (*x* = 0.00, 0.025, 0.050, 0.100) solid solution. This model places oxygen atoms at three different Wyckoff positions: O1 at 18*h*, O2 at 9*e*, and O3 at 36*i*. The barium atoms, Ba1 and Ba2, were placed at the 6*c* and 3*a* sites, respectively. Nb^5+^ and Mo^6+^ were distributed across both 6*c* Wyckoff positions (M1 and M2), in a 1:1 ratio on each site. The Ba and O1 fractional occupancies refined to within ±1% of full occupancy, so were fixed at 1.0. Due to the low atomic number of oxygen, XRD is not able to model the oxygen atoms in the structure with the same accuracy and precision as the metal atoms. Therefore, the fractional occupancies of the O2 and O3 site were fixed at their initial values. The atomic displacement parameters, *U*, were modeled isotropically (*U*_iso_) for all atoms; anisotropic modeling (*U*_aniso_) resulted in the divergence of the refinement.

For the refinement of Ba_3_Nb_1–*x*_Ta_*x*_MoO_8.5_ (*x* = 0.025, 0.050. 0.100), tantalum was incorporated into the model. Initially, Ta^5+^ was distributed equally across the M1 and M2 sites (as Ta1 and Ta2, respectively), in place of Nb^5+^, e.g., for *x* = 0.10, 0.05 was substituted on the M1 site and 0.05 on the M2 site. The M1 and M2 fractional occupancies (*g*) were refined using a *g*(M1) = −*g*(M2) constraint. However, the occupancy of Ta2 refined to either zero or negative values. Therefore, all refinements were repeated with the Ta^5+^ placed exclusively on the Ta1 site, with its fractional occupancy fixed. In further corroboration, if the Ta was distributed equally on the M1 and M2 sites for Ba_3_Nb_0.9_Ta_0.1_MoO_8.5_, χ^2^ and *R*_WP_ were equal to 3.336 and 10.71%, respectively. Upon refining the Ta occupancy (where Ta is observed to occupy the M1 site only), χ^2^ and *R*_WP_ are reduced to 3.299 and 10.65%, respectively.

**Figure 2:**
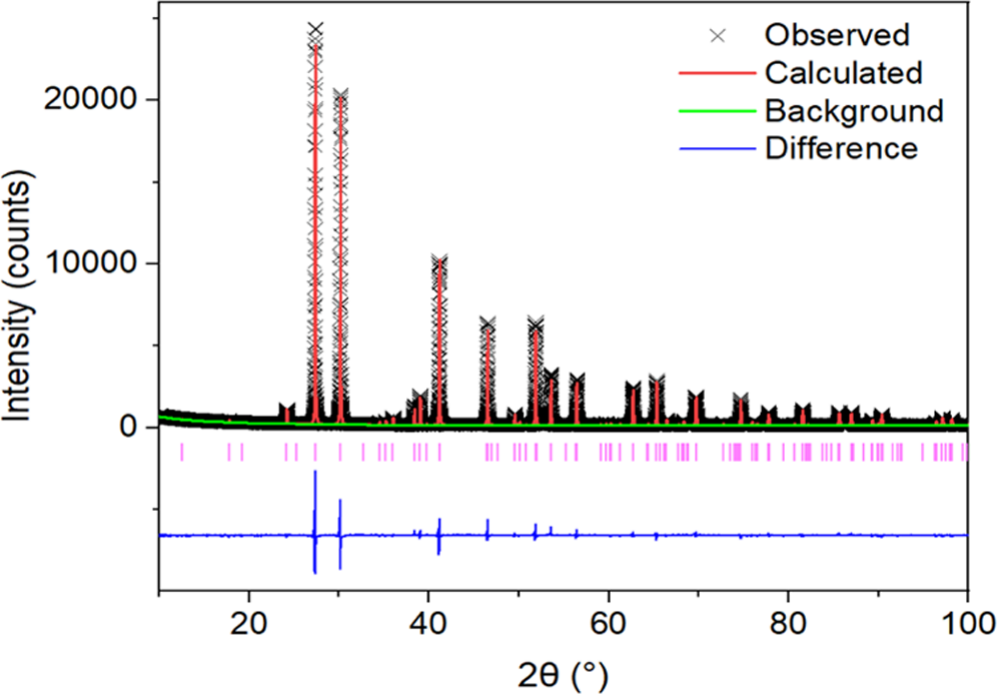
Rietveld refinement fit to the *R*3̅*mH* model of Ba_3_Nb_0.9_Ta_0.1_MoO_8.5_ using XRD data. Black crosses represent the observed data, while the red, green, and blue lines show the Rietveld fit, refined background function, and difference between the observed and calculated patterns, respectively. The pink vertical bars show the reflection positions.

A good Rietveld fit was obtained for all compositions, as evidenced by the statistical parameters reported in Table S2. The Rietveld refinement fit to the XRD pattern of Ba_3_Nb_0.9_Ta_0.1_MoO_8.5_ is displayed in Figure 2; the Rietveld refinement fits for the rest of the series can be found in the Supporting Information (Figure S2). All diffraction patterns could be fitted using the same hexagonal space group *R*3̅*mH*, and no change in symmetry was observed upon Ta doping. An overall increase in the unit cell parameters is observed with increasing Ta content (Table S2), although there is no clear trend with *x*.

Ta substitution increases the total occupancy of the M1 site and hence causes a decrease in the M2 occupancy. It was not possible to determine the average number of tetrahedra in the structure because the oxygen site occupancies were fixed, as discussed earlier. Significant anisotropic peak broadening was observed in the X-ray diffraction patterns for all *x*. This broadening is caused by strain in the lattice. A good fit was obtained using the method previously reported by Stephens where anisotropic broadening of the diffraction peaks is modeled by refining the two independent microstrain covariance parameters, *S*_400_ and *S*_202_.^39^

### AC Impedance Spectroscopy

**Figure 3:**
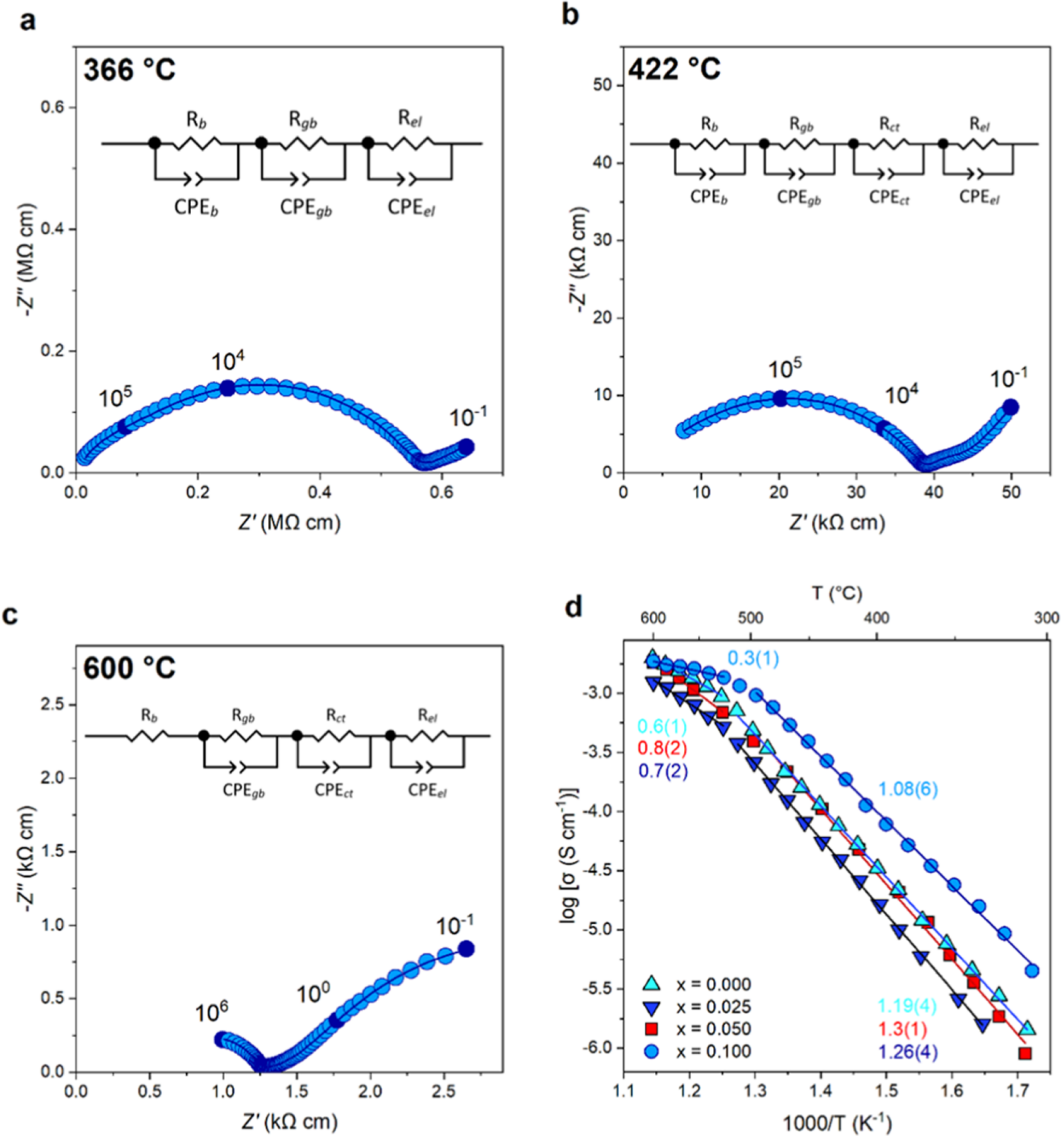
AC Impedance spectroscopy and conductivity of Ba_3_Nb_0.9_Ta_0.1_MoO_8.5_. Complex impedance plots of Ba_3_Nb_0.9_Ta_0.1_MoO_8.5_ recorded under dry air conditions at (a) 366 °C, (b) 422 °C, and (c) 600 °C. The filled circles and their associated labels indicate selected frequencies. The dark blue line represents the equivalent circuit fitting. (d) Arrhenius plot of the bulk ionic conductivities of the Ba_3_Nb_1–*x*_Ta*_x_*MoO_8.5_ series (*x* = 0.00, 0.025, 0.050, 0.100), and the colored numbers represent the activation energy.

The electrical properties of the Ba_3_Nb_1–*x*_Ta_*x*_MoO_8.5_ (*x* = 0.00, 0.025, 0.050, 0.100) series were measured using AC impedance spectroscopy. The resulting complex impedance plots resemble those previously reported for the parent compound, Ba_3_NbMoO_8.5_;^17^ two arcs at high (10^5^ Hz) and intermediate (10^3^ Hz) frequencies represent the bulk (10^–12^ F cm^–1^) and grain boundary (10^–11^–10^–8^ F cm^–1^) responses, respectively. At higher temperatures, a Warburg electrode response in the low-frequency region is characteristic of ionic diffusion in a material with partially blocking electrodes (Figure 3).^40^

The grain boundary arc becomes increasingly depressed as the Ta content increases and all compositions show very poor resolution between the bulk (∼1 × 10^–11^ F cm^–1^) and grain boundary (∼6 × 10^–11^ F cm^–1^) responses due to their similar time constants (τ = *RC*). A pronounced Warburg response is observed for all compositions. Furthermore, the *x* = 0.1 composition exhibits an additional feature between the grain boundary arc and the Warburg response, which can be attributed to charge transfer to and from the oxide ions at the electrode–ceramic interface, as previously reported for V-doped Ba_3_NbMoO_8.5_.^16^

Total resistivity values (*R*_b_ + *R*_gb_) were extracted from the low-frequency intercept of the grain boundary arc and the resulting total conductivity values are presented in the Arrhenius plot in Figure S3. Compared to the parent compound, the Ta-doped compositions with *x* = 0.025 and 0.050 show slightly lower total conductivities, whereas the composition with *x* = 0.1 exhibits an increase in total conductivity; at 600 °C, the total conductivity of Ba_3_Nb_0.9_Ta_0.1_MoO_8.5_ is 8.4 × 10^–4^ S cm^–1^, while Ba_3_NbMoO_8.5_ has a total conductivity of 5.4 × 10^–4^ S cm^–1^.

Resistivity and capacitance values were extracted from the impedance data using equivalent circuit fitting. A resistor in parallel with a constant phase element can be used to model the response of a material to an applied voltage. The overall material response was modeled using multiple RC elements in series to account for the individual responses, i.e., the bulk, grain boundary, charge transfer, and electrode responses. Figure 3 displays the three distinct equivalent circuits that were used to model the data at different temperatures. RC elements for the bulk (*R*_b_-CPE_b_), grain boundary (*R*_gb_-CPE_gb_), and electrode (*R*_el_-CPE_el_) responses were used to model the low-temperature data. At 422 °C, a feature associated with charge transfer to and from the oxide ions at the electrode–ceramic interface appears between the grain arc and the Warburg response. This was modeled with the RC element *R*_ct_-CPE_ct_. For temperatures above 436 °C, the bulk signal moves outside the frequency range of the scan; therefore in Figure 3c, the RC element for the bulk response was substituted by a resistor in series. The goodness of fit was evaluated by the match between the calculated and observed spectra, and the physical plausibility of the fitting parameters. As presented in the Arrhenius plot (Figure 3d), the addition of Ta^5+^ (*x* = 0.025) initially causes a decrease in conductivity in comparison to the parent compound. This conductivity increases upon further Ta^5+^ doping (*x* = 0.050) and becomes larger than the parent compound for *x* = 0.100. A comparison of the bulk conductivity of Ba_3_Nb_0.9_Ta_0.1_MoO_8.5_ with Ba_3_Nb_0.9_V_0.1_MoO_8.5_ and other members of the Ba_3_M′M″O_8.5_ family is shown in Figure S4. At lower temperatures (<500 °C), the bulk conductivity of Ba_3_Nb_0.9_Ta_0.1_MoO_8.5_ is higher than the parent compound; at 300 °C, it exhibits a bulk conductivity (3.8 × 10^–6^ S cm^–1^) 4 times that of the parent (9.4 × 10^–7^ S cm^–1^), with this disparity decreasing as temperature increases so that at 600 °C, both Ba_3_Nb_0.9_Ta_0.1_MoO_8.5_ and Ba_3_NbMoO_8.5_ present a bulk conductivity of ∼2.0 × 10^–3^. This convergence is the result of a lowering of activation energies above 500 °C, in accordance with the more favorable oxide ion conduction pathways generated by the loss of water and subsequent oxygen population rearrangement. The same behavior is observed for the Ba_3_Mo_1–*x*_Nb_1+*x*_O_8.5–*x*/2_ series, where the conductivities of the solid solution converge at higher temperatures, suggesting that the thermal structural rearrangement results in comparable ratios of tetrahedra/octahedra between the compositions.^25^

### Structure Refinement of Ba_3_Nb_0.9_Ta_0.1_MoO_8.5_ by Neutron Powder Diffraction

XRD is unsuitable for accurately determining the location of oxygen atoms within the structure and hence to determine why the *x* = 0.1 composition presents a higher low-temperature conductivity to *x* = 0.0 neutron diffraction data were collected. The same model^16^ used for refinement from the XRD data was used as a starting model for the Rietveld refinement from time-of-flight neutron powder diffraction data of Ba_3_Nb_0.9_Ta_0.1_MoO_8.5_. Again, Nb^5+^ and Mo^6+^ were distributed across both 6*c* Wyckoff positions (M1 and M2), in a 1:1 ratio on each site. In accordance with the X-ray refinement, the Ta^5+^ was only added to the M1 site (Ta1). The M1 and M2 fractional occupancies (*g*) were refined using a *g*(M1) = -*g*(M2) constraint, while O2 and O3 used a *g*(O2) = −0.25*g*(O3) constraint. Ba and O1 fractional occupancies refined to within ±1% of full occupancy and were therefore fixed at 1.0. The atomic displacement parameters, *U*, were modeled anisotropically (*U*_aniso_) for all atoms except the highly disordered O3 position which was modeled isotropically (*U*_iso_). The *U* parameters for the M1 and M2 sites were constrained together to prevent unwanted divergence. A structural model with M2 on a split 6*c* Wyckoff position was employed.^19^

**Figure 4:**
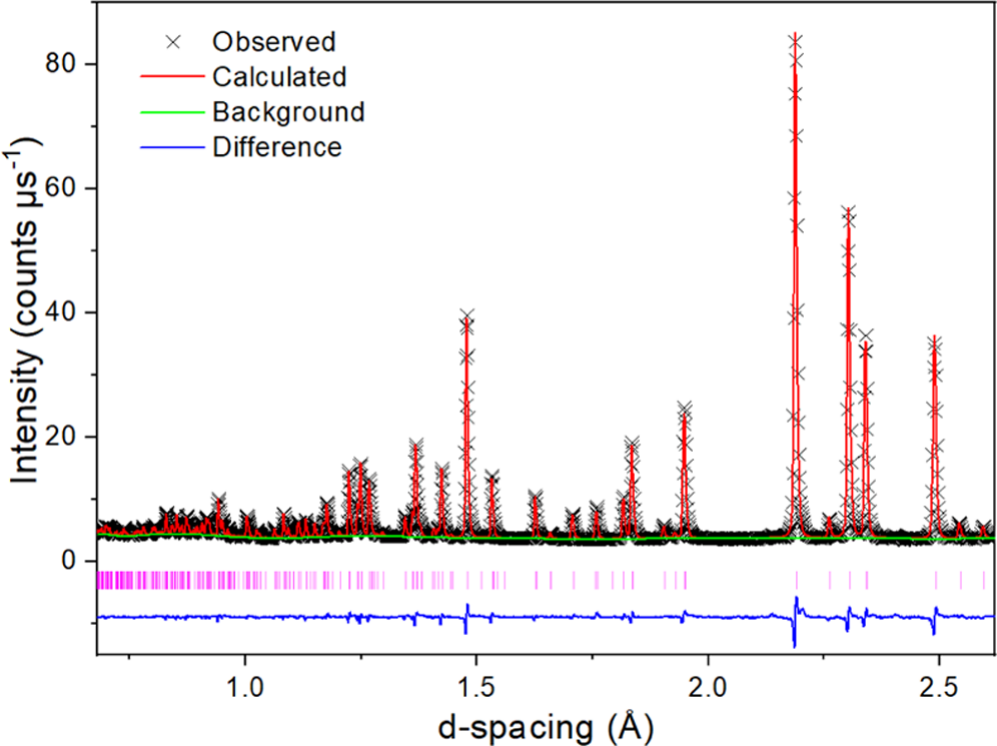
Fitted neutron diffraction histogram for Ba_3_Nb_0.9_Ta_0.1_MoO_8.5_ with TOF neutron data from the 145° detector bank of the Polaris diffractometer. Black crosses represent the observed data, while the red, green, and blue lines show the Rietveld fit, refined background function, and difference between the observed and calculated patterns, respectively. The pink vertical bars show the reflection positions.

An excellent fit was achieved between the calculated and observed histograms (Figures 4 and S4) in the space group *R*3̅*mH* as reported for other members of the Ba_3_M′M″O_8.5_ family (M′ = Nb^5+^, V^5+^; M″ = Mo^6+^, W^6+^).^16,17,26^ The statistical parameters obtained were χ^2^ = 4.005, *R*_p_ = 1.90%, *R*_wp_ = 1.21%. The refined atomic parameters are shown in Table 1. The unit cell parameters *a* = 5.92266(6) Å and *c* = 21.0858(2) Å match up well with the results from the Rietveld refinement from XRD data for the corresponding *x* = 0.1 composition (Table S2), and both neutron and X-ray refinements show larger unit cell parameters than the corresponding parent compound.

**Table 1:** Refined Atomic Parameters from Rietveld Fit to the Crystallographic Model of Ba_3_Nb_0.9_Ta_0.1_MoO_8.5_ from Time-of-Flight Neutron Powder Diffraction Data

atom	site	fraction	*x*	*y*	*z*	*U*_11_ = *U*_22_	*U*_33_	*U*_12_
Ba1	3*a*	1	0	0	0	0.0165(4)	0.0124(6)	0.0082(2)
Ba2	6*c*	1	0	0	0.20710(6)	0.0162(2)	0.0428(6)	0.0081(1)
Nb1	6*c*	0.4004(4)	0	0	0.39832(4)	0.0093(2)	0.0324(4)	0.00463(8)
Mo1	6*c*	0.4447(4)	0	0	0.39832(4)	0.0093(2)	0.0324(4)	0.00463(8)
Ta1	6*c*	0.0504(4)	0	0	0.39832(4)	0.0093(2)	0.0324(4)	0.00463(8)
Nb2	6*c*	0.0496(4)	0	0	0.5242(2)	0.0093(2)	0.0324(4)	0.00463(8)
Mo2	6*c*	0.0553(4)	0	0	0.5242(2)	0.0093(2)	0.0324(4)	0.00463(8)
O1	18*h*	1	0.17313(4)	0.82686(4)	0.10389(2)	0.0246(2)	0.0223(3)	0.0185(2)
							0.0011(1)^b^	-0.0011(1)^c^
O2	9*e*	0.503(2)	0.5	0	0	0.0248(5)	0.0371(9)	0.0280(5)
							0.0199(4)^b^	0.0398(9)^c^
O3	36*i*	0.0831(4)	0.0929(4)	0.0784(7)	0.3202(1)	0.030(1)^a^		

a *U*_iso_ (Å^2^).

b *U*_13_.

c *U*_23_. *U_ij_* (in Å^2^) represents anisotropic displacement parameters.

The Ta-doped material shows an increase in overall M1 site occupancy (which agrees with the trend observed for the X-ray series) but a decrease in occupancy of the tetrahedra-forming O3 site (0.092 to 0.083), causing the percentage of tetrahedra to fall from 54.2% to 49.8%. This suggests that Ta doping leads to more octahedra on the M1 site, in accordance with the preference of Ta^5+^ ions for octahedral coordination.^28^ The ratio of O2/O3 site occupancies and hence percentage of tetrahedra in the structure is regarded as an important factor dictating the conductivity of the Ba_3_M′M″O_8.5_ phases (M′ = Nb^5+^, V^5+^; M″ = Mo^6+^, W^6+^),^16,17,26^ as a higher proportion of M1O_4_ tetrahedra provides an increased amount of favorable pathways for oxide ion conduction. However, this is only true if other supplementary factors remain equal; M1–O3 distance, polyhedral distortion, and M2 site occupancy are also key to achieving high ionic conductivity. Therefore, the higher conductivity but lower proportion of tetrahedra in the *x* = 0.1 sample suggests that there are other factors that must be considered.

Table S3 shows a comparison of the bond lengths of Ba_3_NbMoO_8.5_, Ba_3_Nb_0.9_V_0.1_MoO_8.5_, and Ba_3_Nb_0.9_Ta_0.1_MoO_8.5_. The bond angles are shown in Table S4. The M1–O3 bond, in particular, is significantly longer for Ba_3_Nb_0.9_Ta_0.1_MoO_8.5_ than all of the other Ba_3_M′M″O_8.5_ phases. The distance between the M1 position and the mobile oxygen species is known to influence ionic conduction and commonly occurs due to the out-of-center displacement (*D*) of the M1 atom away from the O2/O3 sites. The PIEFACE software package was employed to analyze the minimum bounding ellipsoid (i.e., the smallest volume ellipsoid able to contain all atoms of the coordination polyhedron). For further elaboration, see the Supporting Information. It was observed that the value of *D* remains relatively unchanged for the M1 site (for both tetrahedra and octahedra) upon Ta doping, whereas the M2 site sees a shift toward the center of the polyhedra (Table S5). Therefore, the longer M1–O3 bond length for Ba_3_Nb_0.9_Ta_0.1_MoO_8.5_ is not a result of displacement.

Polyhedral distortion can also result in significant changes in bond length and was therefore quantified using PIEFACE. The minimum bounding ellipsoid has three radii (*R*_1_, *R*_2_, and *R*_3_) with a mean value of *R*. The standard deviation, σ(*R*), is equal to , where σ^2^(*R*) is the variance, i.e., the average of the squared differences from the mean. Therefore, the standard deviation is used as a measure of the polyhedral distortion. The σ(*R*) value of the M1O_4_ tetrahedra is significantly higher in the Ta-doped material than the parent compound, increasing from 0.05362 to 0.06486 Å, showing that the tetrahedra become more distorted with Ta doping. The M2O_6_ octahedra also become more distorted (with σ(*R*) increasing from 0.12934 to 0.13204 Å), but the M1O_6_ octahedra remain unchanged; the Ta-doped structure is more distorted overall than the parent compound. Therefore, the longer M1–O3 bond length observed in Ba_3_Nb_0.9_Ta_0.1_MoO_8.5_ is most likely a consequence of the larger distortion of the MO*_x_* polyhedra as a result of the greater polarizability of Ta^5+^ compared to Nb^5+^.^41^ Increasing the distance between the M1 and O2/O3 sites has previously been shown to decrease the energy barrier to migration by lowering the motional enthalpy required for the movement of oxide ions.^42^ Therefore, the increase in M1–O3 bond length with Ta doping supports the enhanced conductivity that is observed. The increased distortion of the M1O_4_ and M2O_6_ octahedra is most likely also the origin of the lattice expansion, which is observed upon substitution of Ta^5+^ for Nb^5+^.

The shape of the variable coordination polyhedra can be described using the ellipsoid shape parameter, *S* (Table S5). In addition to being more distorted, the ellipsoid defined for the M1O_4_ tetrahedra becomes more prolate (axially stretched, 1 ≥ *S* > 0), which correlates with the increased M1–O3 bond length. Both M1O_6_ and M2O_6_ octahedra remain relatively unchanged (but still oblate i.e., axially compressed, 0 > *S* ≥ −1).

These results suggest there are competing factors influencing the overall bulk ionic conductivity of Ba_3_M′M″O_8.5_ phases. As Ta is introduced into the structure, we see a decrease in the number of tetrahedra and an associated drop in conductivity. Table S3 also shows that the O2–O2 and O2–O3 distances increase with lattice expansion. Given that *x* = 0.025 and *x* = 0.050 have larger cell volumes than *x* = 0.100, it is likely that the O2–O2 and O2–O3 distances are longer, which would also explain the reduced oxide ionic conductivity.

However, as the proportion of Ta increases, the M1–O3 bond elongates and the conductivity increases with *x* until it surpasses that of the parent compound for *x* = 0.1. This suggests that once the M1–O3 bond length reaches a critical point, it facilitates a net increase in the overall conductivity, even though the ratio of tetrahedra:octahedra has decreased with respect to the parent compound and the O2–O2 and O2–O3 distances are longer. The seemingly diminished role of the tetrahedra:octahedra ratio is not unprecedented; in the recent work on Ba_3_VWO_8.5_,^15^ the structure is shown to have an extraordinarily high number of highly distorted tetrahedra, but it exhibits relatively low levels of ionic conductivity due to the lack of an occupied M2 site. This disrupts the three-dimensional (3D) network within the structure, thereby preventing conduction along the *c*-axis between the two-dimensional palmierite-like layers.

## Conclusions

In summary, we have shown that the low-temperature (<500 °C) ionic conductivity of Ba_3_NbMoO_8.5_ can be significantly enhanced by substituting Nb^5+^ with Ta^5+^. Ba_3_Nb_0.9_Ta_0.1_MoO_8.5_ exhibits the highest low-temperature ionic conductivity among the Ba_3_Nb_1–*x*_Ta_*x*_MoO_8.5_ (*x* = 0.00, 0.025, 0.050, 0.100) series. It may be possible to increase the conductivity further if higher Ta^5+^ doping levels could be achieved. The results indicate that a combination of factors are needed to realize the highest ionic conductivity in the Ba_3_M′M″O_8.5_ family. These include a large unit cell with long M1–O3 bonds, an occupied M2 site, disordered polyhedra (σ(*R*) > 0.06), and a sufficiently high ratio of tetrahedra/octahedra. Co-doping strategies such as the substitution of both V^5+^ and Ta^5+^ for Nb^5+^ could result in even higher oxide ion conductivities than reported to date.

## Supporting Information Available

Rietveld refinement fits, scanning electron microscopy images, and minimum bounding ellipsoid data (PDF)
