# Supplementary material for: Enhanced Oxide Ion Conductivity by Ta Doping of Ba3Nb1–xTaxMoO8.5
Source: Inorg Chem. 2023 Jan 17;62(4):1628–35. doi: 10.1021/acs.inorgchem.2c03943 (PMC9890478; doi:10.1021/acs.inorgchem.2c03943)
Supplement: Supplementary file 1 — ic2c03943_si_001.pdf [file ic2c03943_si_001.pdf]

Supplementary information

for

Enhanced Oxide Ion Conductivity by Ta Doping of $Ba_3Nb_{1-x}Ta_xMoO_{8.5}$

Brent Sherwood,[a] Eve J. Wildman,[a] Ronald I. Smith,[b] and Abbie C. Mclaughlin*[a]

[a]Department of Chemistry, University of Aberdeen, Meston Walk, Aberdeen AB24 3UE,UK. E-mail: a.c.mclaughlin@abdn.ac.uk

[b]ISIS Facility, STFC Rutherford Appleton Laboratory, Harwell Campus, Didcot, OX110QX, UK

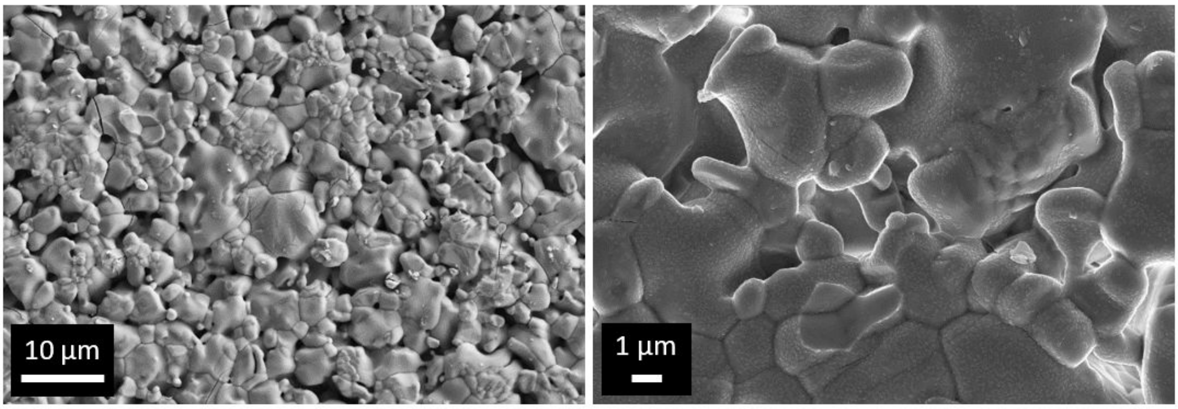

Figure S1. SEM micrographs of the surface of $Ba_3Nb_{0.9}Ta_{0.1}MoO_{8.5}$. Secondary electron (SE) and backscattering electron (BSE) images are shown on the left and right, respectively.

Table S1. EDX spectroscopy data of $Ba_3Nb_{1-x}Ta_xMoO_{8.5}$ series (x = 0.00, 0.025, 0.050, 0.100).

	X			
**Atom**	**0.00**	**0.025**	**0.050**	**0.100**
**O**	8.5	8.5	8.5	8.5
**Nb**	0.9995	0.9775	0.9495	0.8925
**Mo**	1.001	0.9875	0.983	0.997
**Ba**	2.9965	3.027	3.0485	3.03
**Ta**	0	0.0275	0.0535	0.0985

EDX analysis shows the presence of uniformly distributed Nb, Mo, Ba, O and Ta so that the samples are compositionally homogeneous within the spatial resolution of SEM-EDX analysis. The average atomic percentages calculated from the measurements are close to the theoretical values within instrument resolution and standard deviations as shown in Table S1.

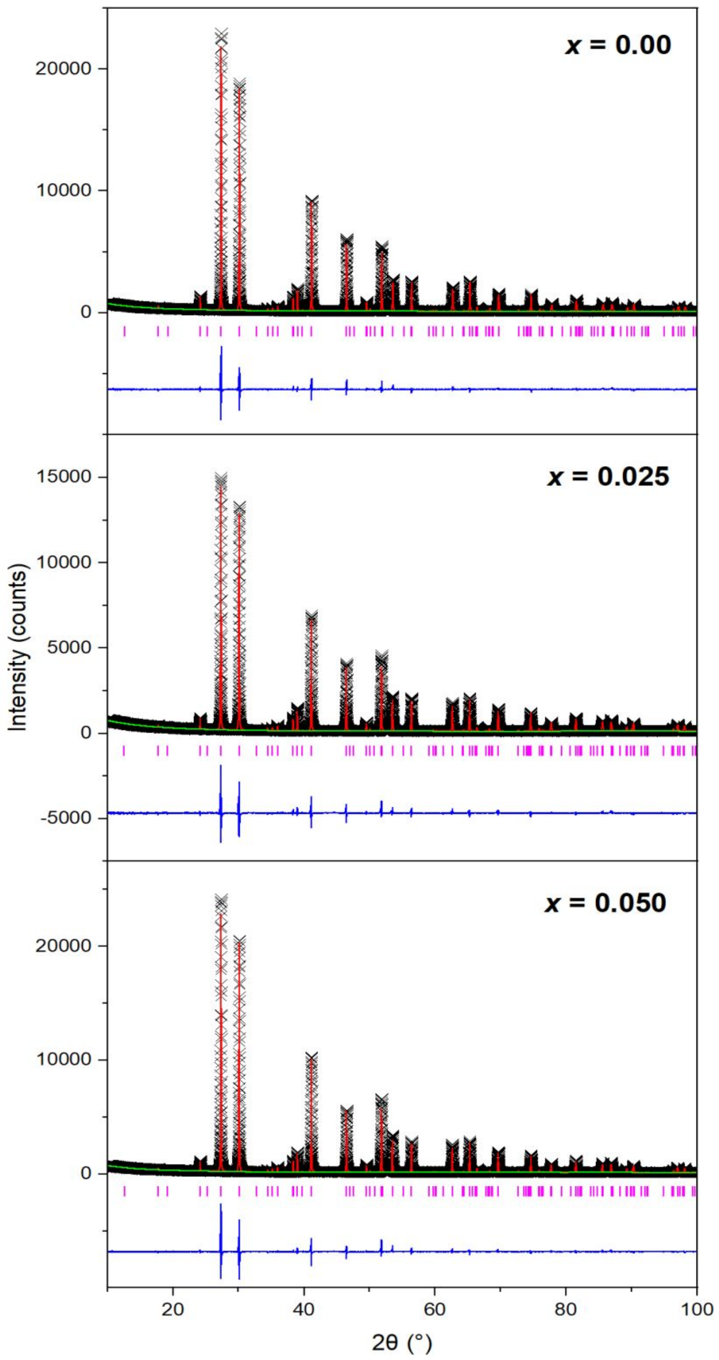

Figure S2. Rietveld refinement fit to the XRD data of $Ba_3Nb_{1-x}Ta_xMoO_{8.5}$ (x = 0.00, 0.025 and 0.050). Black crosses represent the observed data, while the red, green, and blue lines show the Rietveld fit, background and difference between the observed and calculated patterns, respectively. The pink vertical bars show the reflection positions.

Table S2. Refined atomic parameters and statistical parameters from Rietveld fit of the powder XRD data of $Ba_3Nb_{1-x}Ta_xMoO_{8.5}$ (x = 0.00, 0.025, 0.050, 0.100).

		***x* = 0.00**	***x* = 0.025**	***x* = 0.050**	***x* = 0.100**
***a* (Å)**		5.91824 (2)	5.92303 (3)	5.92551 (2)	5.92217 (2)
***c* (Å)**		21.08395 (9)	21.0921 (1)	21.08919 (9)	21.08824 (8)
**V (Å^3)**		639.539 (6)	640.822 (7)	641.270 (5)	640.519 (5)
		***x* = 0.00**	***x* = 0.025**	***x* = 0.050**	***x* = 0.100**
**Ba1**<br>(0, 0, 0) 3*a*	U_{iso}	0.0117 (5)	0.0080 (6)	0.0148 (5)	0.0123 (4)
**Ba2**<br>(0, 0, *z*) 6*c*	*z*<br>U_{iso}	0.20835 (4)<br>0.0236 (4)	0.20822 (5)<br>0.0173 (4)	0.20773 (5)<br>0.0254 (4)	0.20795 (4)<br>0.0234 (4)
**M1**<br>(0, 0, *z*) 6*c*	*z*<br>**Frac (Nb1)**<br>**Frac (Mo1)**<br>**Frac (Ta1)**<br>U_{iso}	0.39786 (7)<br>0.4495 (8)<br>0.4495 (8)<br>-<br>0.0107 (4)	0.39791 (9)<br>0.438 (1)<br>0.450 (1)<br>0.0125 (0)<br>0.0052 (5)	0.39805 (8)<br>0.4316 (9)<br>0.4566 (9)<br>0.025 (0)<br>0.0139 (4)	0.39765 (7)<br>0.4033 (8)<br>0.4532 (8)<br>0.05 (0)<br>0.0125 (4)
**M2**<br>(0, 0, *z*) 6*c*	*z*<br>**Frac (Nb2)**<br>**Frac (Mo2)**<br>**Frac (Ta2)**<br>U_{iso}	0.5192 (4)<br>0.0505 (8)<br>0.0505 (8)<br>-<br>0.0107 (4)	0.5183 (5)<br>0.050 (1)<br>0.050 (1)<br>0<br>0.0052 (5)	0.5191 (5)<br>0.0434 (9)<br>0.0434 (9)<br>0<br>0.0139 (4)	0.5178 (4)<br>0.0468 (8)<br>0.0468 (8)<br>0<br>0.0125 (4)
**O1**<br>(x, $\bar{x}$, z) 18*h*	*x*<br>*y*<br>*z*<br>U_{iso}	0.1698 (4)<br>0.8302 (4)<br>0.1043 (2)<br>0.019 (1)	0.1674 (5)<br>0.8325 (5)<br>0.1054 (2)<br>0.017 (1)	0.1704 (5)<br>0.8295 (5)<br>0.1048 (2)<br>0.026 (1)	0.1694 (4)<br>0.8306 (4)<br>0.1042 (2)<br>0.020 (1)
**O2**<br>(0.5, 0, 0) 9*e*	**Frac**<br>U_{iso}	0.467 (0)<br>0.019 (1)	0.467 (0)<br>0.017 (1)	0.467 (0)<br>0.026 (1)	0.467 (0)<br>0.020 (1)
**O3**<br>(*x*, *y*, *z*) 36*i*	*x*<br>*y*<br>*z*<br>**Frac**<br>U_{iso}	0.082 (8)<br>0.095 (5)<br>0.3199 (7)<br>0.092 (0)<br>0.019 (1)	0.08 (2)<br>0.122 (4)<br>0.3205 (9)<br>0.092 (0)<br>0.017 (1)	0.081 (7)<br>0.082 (6)<br>0.3240 (8)<br>0.092 (0)<br>0.026 (1)	0.111 (5)<br>0.115 (4)<br>0.3203 (8)<br>0.092 (0)<br>0.020 (1)
		***x* = 0.00**	***x* = 0.025**	***x* = 0.050**	***x* = 0.100**
χ^2		3.535	3.630	3.995	3.299
**R_p (%)**		8.02	8.61	8.61	8.09
**R_{wp} (%)**		10.93	11.70	11.71	10.65
**RF2 (%)**		11.67	14.63	11.51	11.80

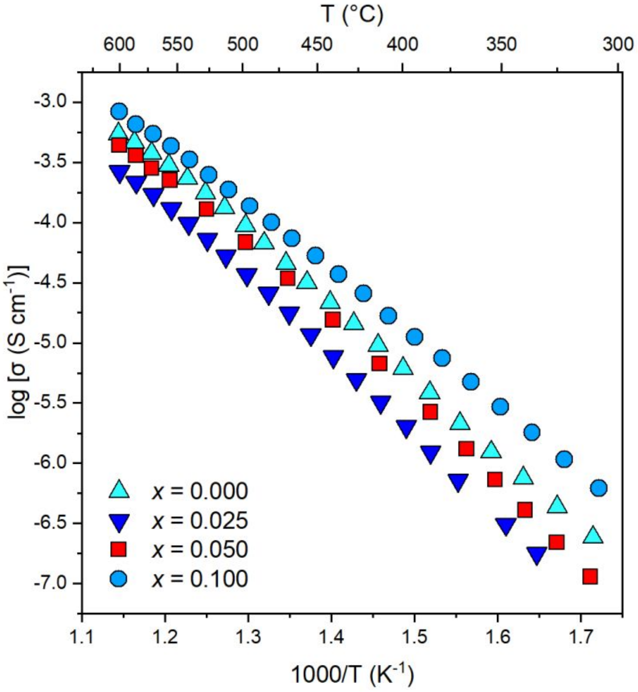

Figure S3. Arrhenius plot of the total ionic conductivity of $Ba_3Nb_{1-x}Ta_xMoO_{8.5}$ (x = 0.00, 0.025, 0.050, 0.100).

Table S3. Selected bond lengths calculated by Rietveld refinement of powder neutron diffraction data of $Ba_3NbMoO_{8.5}$,[1] $Ba_3Nb_{0.9}Ta_{0.1}MoO_{8.5}$ and $Ba_3Nb_{0.9}V_{0.1}MoO_{8.5}$.[1]

**Bond length (Å)**	**$Ba_3NbMoO_{8.5}$**	**$Ba_3Nb_{0.9}Ta_{0.1}MoO_{8.5}$**	**$Ba_3Nb_{0.9}V_{0.1}MoO_{8.5}$**
**Ba1-O1 x 6**	2.8171 (6)	2.8202 (4)	2.8064 (4)
**Ba1-O2 x 6**	2.95986 (4)	2.96133 (3)	2.96009 (3)
**Ba1-O3 x 12**	2.955 (4)	2.961 (2)	2.987 (3)
**Ba2-O1 x 3**	2.802 (2)	2.809 (1)	2.8102 (14)
**Ba2-O1 x 6**	2.9983 (5)	2.9993 (2)	3.0007 (4)
**Ba2-O2 x 3**	3.166 (2)	3.164 (1)	3.1688 (15)
**Ba2-O3 x 6**	2.476 (5)	2.440 (3)	2.481 (3)
**M1-O1**	1.8379 (8)	1.8368 (5)	1.8362 (6)
**M1-O2**	2.1926 (8)	2.1909 (5)	2.1942 (7)
**M1-O3**	1.706 (5)	1.725 (3)	1.687 (3)
**M2-O1**	1.857 (3)	1.834 (2)	1.848 (3)
**M2-O1**	2.429 (4)	2.462 (3)	2.474 (5)
**O2-O2**	2.95986 (4)	2.96133 (4)	2.96009 (3)
**O2-O3**	1.331 (5)	1.373 (4)	1.354 (4)

Table S4. Selected bond angles calculated by Rietveld refinement of powder neutron diffraction data of $Ba_3NbMoO_{8.5}$,[1] $Ba_3Nb_{0.9}Ta_{0.1}MoO_{8.5}$ and $Ba_3Nb_{0.9}V_{0.1}MoO_{8.5}$.[1]

Bond angle (°)	$Ba_3NbMoO_{8.5}$	$Ba_3Nb_{0.9}Ta_{0.1}MoO_{8.5}$	$Ba_3Nb_{0.9}V_{0.1}MoO_{8.5}$
**O1-M1-O1**	101.76 (5)	101.57 (3)	102.26 (4)
**O1-M1-O2**	85.95 (2)	86.00 (1)	85.671 (14)
	167.58 (7)	167.83 (4)	167.13 (6)
**O1-M1-O3**	99.8 (1)	100.31 (7)	100.38 (9)
	116.5 (2)	117.7 (1)	116.15 (16)
	130.7 (2)	129.5 (1)	129.42 (14)
**O2-M1-O2**	84.90 (4)	85.04 (2)	84.84 (4)
**O1-M2-O1**	71.9 (1)	70.6 (1)	70.59 (16)
	92.27 (6)	91.81 (6)	92.10 (9)
	100.3 (2)	101.8 (2)	101.4 (3)
	160.2 (2)	158.3 (2)	158.5 (3)

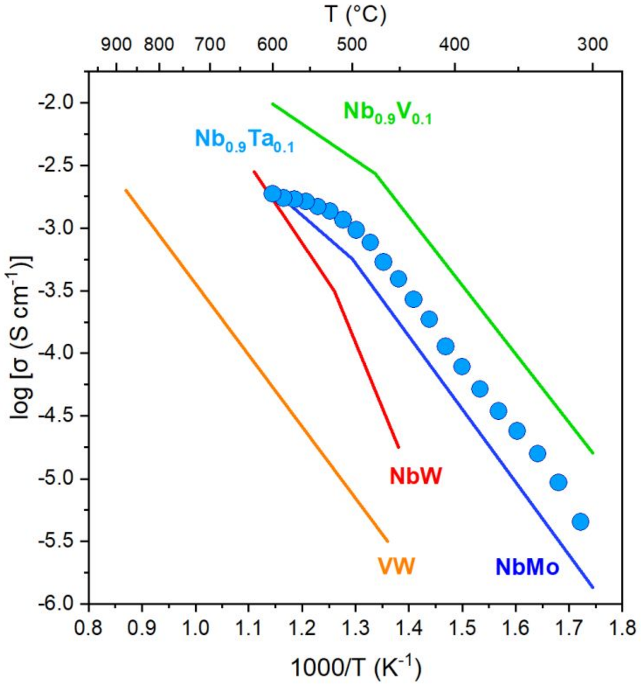

Figure S4. Arrhenius plot comparing the bulk conductivity of $Ba_3MoNb_{0.9}Ta_{0.1}O_{8.5}$ (blue circles) with other members of the $Ba_3M'M''O_{8.5}$ family: $Ba_3MoNb_{0.9}V_{0.1}O_{8.5}$ (green)[1], $Ba_3MoNbO_{8.5}$ (dark blue)[1], $Ba_3NbWO_{8.5}$ (red)[2], and $Ba_3VWO_{8.5}$ (orange)[3].

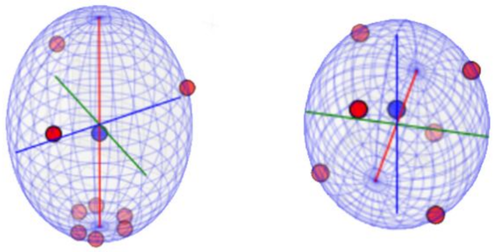

Figure S5. Examples of minimum-bounding ellipsoids for $M1O_4$ (left) and $M1O_6$ (right) polyhedra in $Ba_3MoNb_{0.9}Ta_{0.1}O_{8.5}$, calculated using PIEFACE. The red (R_1), green (R_2), and blue (R_3) lines correspond to the three principal radii of the ellipsoid (where $R_1 \geq R_2 \geq R_3$). The small blue and red spheres represent the central metal cation and surrounding oxide anions, respectively.

Table S5. Results from ellipsoidal analysis of coordination polyhedra using PIEFACE. [4] The standard deviation, $\sigma(R)$, of the ellipsoid radii (R_1, R_2, R_3) quantifies polyhedral distortion. The ellipsoid shape parameter, $S = R_3/R_2 - R_2/R_1$, has a range of $-1 \leq S \leq 1$ where axially compressed (oblate), axially stretched (prolate), and spherical ellipsoids have $S < 0$, $S > 0$, and $S = 0$, respectively. The value of D quantifies the displacement of the central atom relative to the ellipsoid centre.

**Parameter**	**Coordination**	**$Ba_3NbMoO_{8.5}$**	**$Ba_3Nb_{0.9}Ta_{0.1}MoO_{8.5}$**	**$Ba_3Nb_{0.9}V_{0.1}MoO_{8.5}$**
σ(R) (Å)	M1O4	0.05362	0.06486	0.04194
	M1O6	0.07578	0.07525	0.08111
	M2O6	0.12934	0.13204	0.13831
S	M1O4	0.0608	0.0728	0.0481
	M1O6	-0.0782	-0.0777	-0.0836
	M2O6	0.1198	0.1222	0.1267
D (Å)	M1O4	0.1622	0.1613	0.1617
	M1O6	-0.3192	-0.318	-0.3237
	M2O6	-0.4637	-0.5092	-0.5064

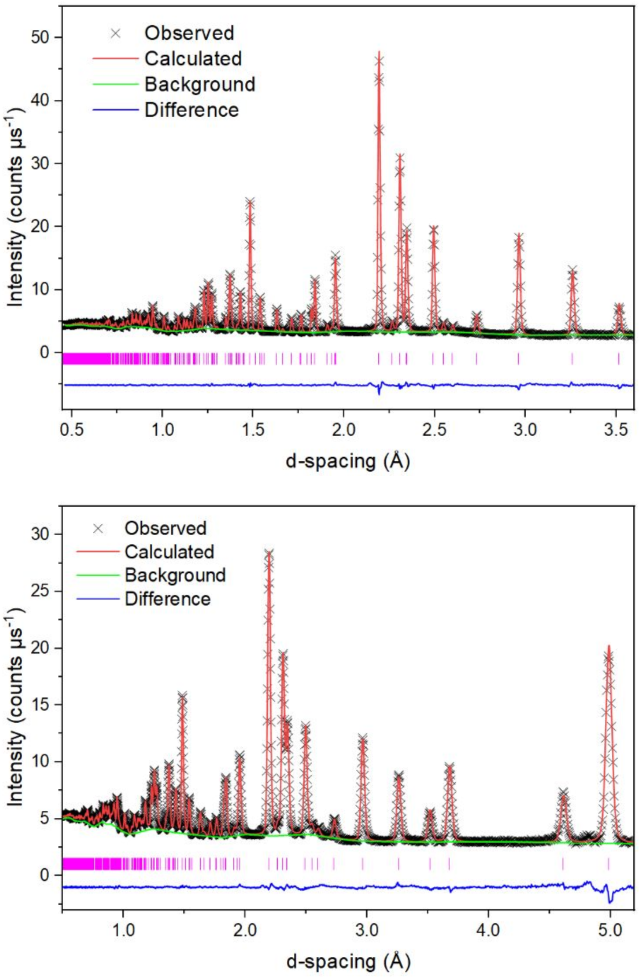

Figure S6. Fitted neutron diffraction histograms for $Ba_3Nb_{0.9}Ta_{0.1}MoO_{8.5}$ with TOF neutron data from the 90° (top) and 50° (bottom) detector banks of the Polaris diffractometer. Black crosses represent the observed data, while the red, green, and blue lines show the Rietveld fit, background and difference between the observed and calculated patterns, respectively. The pink vertical bars show the reflection positions.

Table S6: Crystallographic data table

**Source**	Neutron (time of flight)
**Chemical formula**	$Ba_3Nb_{0.9}Ta_{0.1}MoO_{8.5}$
**Formula weight**	745.6361
**Temperature (K)**	300
**Crystal System**	Trigonal
**Space group**	$R\bar{3}m$ H (no. 166)
***a* (Å)**	5.92266(6)
***c* (Å)**	21.0858(2)
**V (Å^3)**	640.5331(7)
Z	3
***d*-space range (Å)**	0.47 – 5.241
χ^2	4.005
R_P	1.90
R_{WP}	1.21
**Definition of R factors**	$R_p = \Sigma\|y_i(obs) - y_i(calc)\|/\Sigma y_i(obs)$;<br>$R_{wp} = \{\Sigma w_i[y_i(obs) - y_i(calc)]^2/\Sigma w_i y_i(obs)^2\}^{1/2}$

References

(1) Fop, S.; McCombie, K.; Smith, R. I.; McLaughlin, A. C. Enhanced Oxygen Ion Conductivity and Mechanistic Understanding in $Ba_3Nb_{1-x}V_xMoO_{8.5}$. *Chemistry of Materials* **2020**, *32* (11), 4724–4733.

(2) McCombie, K. S.; Wildman, E. J.; Fop, S.; Smith, R. I.; Skakle, J. M. S.; McLaughlin, A. C. The Crystal Structure and Electrical Properties of the Oxide Ion Conductor $Ba_3WNbO_{8.5}$. *J. Mater. Chem. A Mater.* **2018**, *6* (13), 5290–5295.

(3) Gilane, A.; Fop, S.; Sher, F.; Smith, R. I.; McLaughlin, A. C. The Relationship between Oxide-Ion Conductivity and Cation Vacancy Order in the Hybrid Hexagonal Perovskite $Ba_3VWO_{8.5}$. *J. Mater. Chem. A Mater.* **2020**, *8* (32), 16506–16514.

(4) Cumby, J.; Attfield, J. P. Ellipsoidal Analysis of Coordination Polyhedra. *Nat. Commun.* **2017**, *8*.
